# Mid-Term Outcomes of Metal-Backed Unicompartmental Knee Arthroplasty Show Superiority to All-Polyethylene Unicompartmental and Total Knee Arthroplasty

**DOI:** 10.1007/s11420-017-9557-5

**Published:** 2017-05-12

**Authors:** Jelle P. van der List, Laura J. Kleeblad, Hendrik A. Zuiderbaan, Andrew D. Pearle

**Affiliations:** 1 Computer Assisted Surgery Center, Department of Orthopaedic Surgery, Hospital for Special Surgery, 535 East 70th Street, New York, NY 10021 USA; 2 Weill Cornell Medical College, New York, NY 10065 USA; 3 Department of Orthopaedic Surgery, Spaarne Hospital, Hoofddorp, The Netherlands

**Keywords:** unicompartmental knee arthroplasty, total knee arthroplasty, outcomes, all-polyethylene, metal-backed

## Abstract

**Background:**

Two commonly used tibial designs for unicompartmental knee arthroplasty (UKA) are all-polyethylene “inlay” and metal-backed “onlay” components. Biomechanical studies showed that the metal baseplate in onlay designs better distributes forces over the tibia but studies failed to show differences in functional outcomes between both designs at mid-term follow-up. Furthermore, no studies have compared both designs with total knee arthroplasty (TKA).

**Questions/Purposes:**

The goal of this study was to compare outcomes of inlay UKA and onlay UKA at mid-term follow-up and compare these with TKA outcomes.

**Methods:**

In this retrospective study, 52 patients undergoing inlay medial UKA, 59 patients undergoing onlay medial UKA, and 59 patients undergoing TKA were included. Western Ontario and McMaster Universities Arthritis Index scores were collected preoperatively and at mean 5.1-year follow-up (range 4.0–7.0 years).

**Results:**

Preoperatively, no differences were observed in patient characteristics or outcome scores. At mid-term follow-up, patients undergoing onlay medial UKA reported significant better functional outcomes than those of inlay medial UKA (92.0 ± 10.4 vs. 82.4 ± 18.7, *p* = 0.010) and when compared to TKA (92.0 ± 10.4 vs. 79.6 ± 18.5, *p* < 0.001) while no significant differences between inlay medial UKA and TKA were noted. No significant differences in revision rates were found.

**Conclusion:**

Functional outcomes following onlay metal-backed medial UKA were significantly better compared to inlay all-polyethylene medial UKA and to TKA. Based on the results of this study and on biomechanical and survivorship studies in the literature, we recommended using metal-backed onlay tibial components for unicompartmental knee arthroplasty.

**Electronic supplementary material:**

The online version of this article (doi:10.1007/s11420-017-9557-5) contains supplementary material, which is available to authorized users.

## Introduction

Unicompartmental knee arthroplasty (UKA) and total knee arthroplasty (TKA) are two reliable treatment options for medial knee osteoarthritis (OA). UKA is increasingly popular [42] and has distinct advantages over TKA including faster recovery [31, 50, 56], better range of motion [26], better function [2, 36, 40, 55, 67], and more cost-effectiveness [13, 18, 44, 63], while TKA has a higher survivorship [1, 28, 38, 57].

**Fig. 1:**
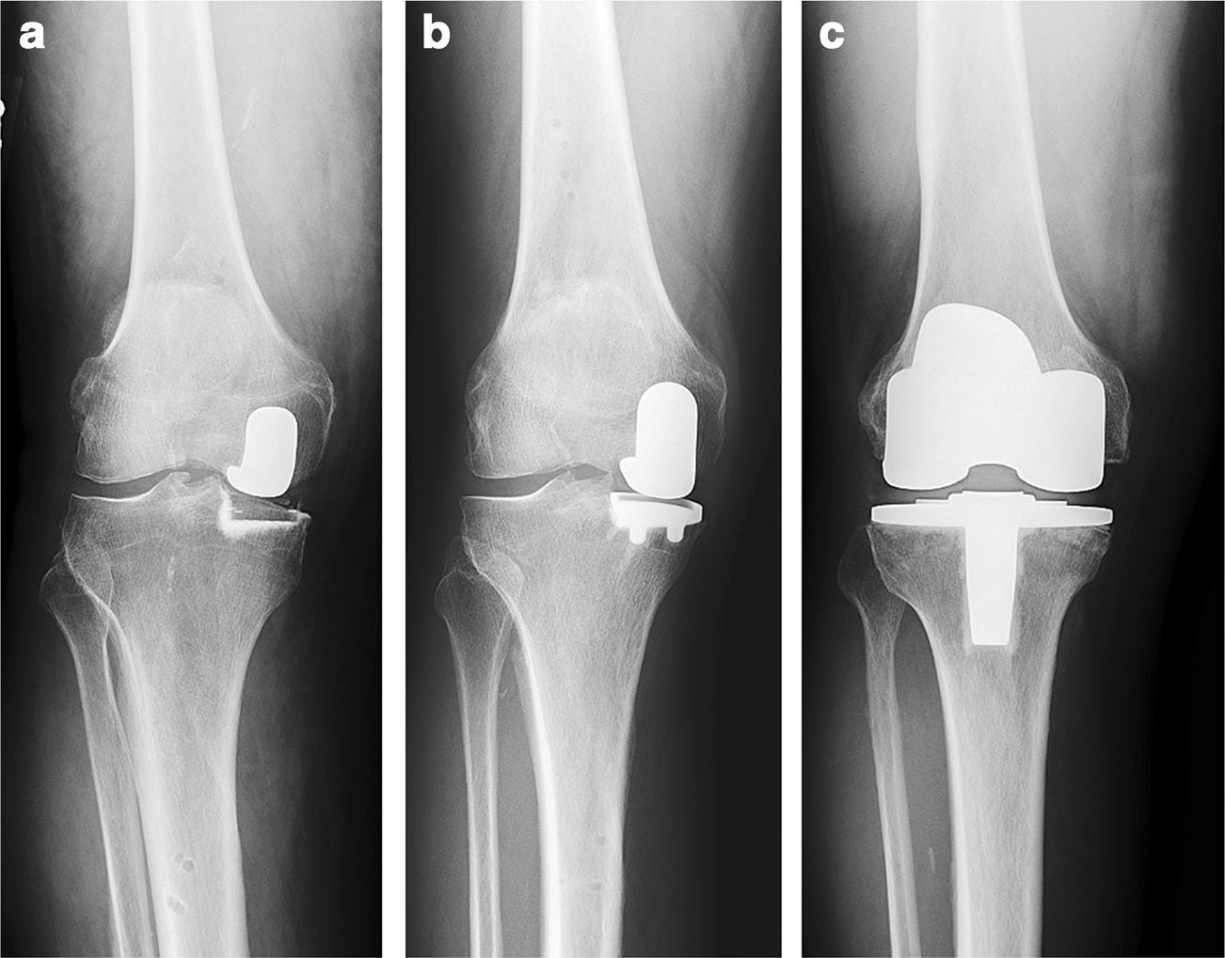
**a** An all-polyethylene “inlay” medial unicompartmental knee arthroplasty. **b** A metal-backed “onlay” medial unicompartmental knee arthroplasty. **c** A total knee arthroplasty.

Two commonly used fixed-bearing UKA tibial components are all-polyethylene “inlay” components and metal-backed “onlay” components. Inlay components are cemented into a carved pocket on the tibial surface and therefore rely more on the subchondral bone (Fig. 1a) whereas onlay components are cemented on top of the flat tibial cut with a metal baseplate and therefore rely on the cortical bone as well as on the subchondral bone (Fig. 1b) [19, 43]. Studies have shown that inlay components have higher peak stress at the tibial surface compared to onlay components [49, 61], which could be explained by the fact that onlay components rest on the cortical bone and that the metal backing distributes forces over the tibia [49, 61]. Perhaps, as a result, a higher incidence of tibial subsidence [3, 66] and revisions are seen with inlay designs [3, 22, 66].

It has further been suggested that this increased stress can cause pain and inferior outcomes [19, 48]. Although one study reported inferior clinical outcomes with inlay components at short-term follow-up, at mid-term follow-up no clear significant or clinically relevant difference between both components is seen in functional outcomes [20, 22]. Because of this discrepancy, the first goal of this study was to compare outcomes of onlay and inlay medial UKA at mid-term follow-up. Robotic-assisted UKA surgery was used which provides tighter control of variables, such as lower leg alignment, gap balancing, and component positioning [14, 32, 35, 43, 45, 52, 53]. Furthermore, although many recent studies have shown superior outcomes of UKA compared to those of TKA [16, 29, 36, 41, 60, 64, 67], none of the studies have, to our knowledge, compared both inlay and onlay designs to TKA within one study. Therefore, the second goal of this study was to compare onlay and inlay designs with TKA to assess if both components are superior to TKA.

## Patients and Methods

In this retrospective study, a search was performed in the digital database of the senior author for patients undergoing medial UKA and TKA between May 2007 and March 2012. Surgical inclusion criteria were unicompartmental medial OA or multicompartmental OA for medial UKA and TKA surgery, respectively. Patients were excluded from the search if they (I) had ACL deficiency or (II) did not undergo robotic-assisted UKA or computer-navigated TKA surgery. A total of 170 patients had minimum 4- and maximum 7-year follow-up, of which 52 underwent inlay medial UKA; 59 onlay medial UKA; and 59 TKA. Of these patients, 116 completed the Western Ontario and McMaster Universities Arthritis Index (WOMAC) questionnaire (36 inlay medial UKA patients, 42 onlay medial UKA patients, and 38 TKA patients). Baseline characteristics are displayed in Table 1.

**Table 1:** Patient demographics of patients undergoing medial UKA and TKA

	MUKA inlay (*n* = 52)	MUKA onlay (*n* = 59)	TKA (*n* = 59)	ANOVA
	Mean (±SD)	Mean (±SD)	Mean (±SD)	*p* value
Age (years)	61.7 (±10.2)	64.6 (±8.7)	64.3 (±7.5)	0.305
BMI (kg/m^2^)	31.3 (±5.7)	29.3 (±6.3)	31.5 (±6.4)	0.220
Gender (M:F)	30:22	31:28	21:38	0.048^*^
OA severity MC (KL)	3.2 (±0.7)	3.1 (±0.8)	3.0 (±0.9)	0.789
OA severity LC (KL)	0.3 (±0.5)	0.6 (±0.7)	1.4 (±1.0)	<0.001^**^
OA severity PFC (KL)	0.9 (±0.2)	0.6 (±0.7)	1.4 (±1.0)	<0.001^**^
Preoperative alignment (°)	6.4 (±4.0)	7.1 (±3.7)	4.3 (±8.2)	0.144
Postoperative alignment (°)	2.9 (±3.3)	2.0 (±2.0)	0.9 (±3.2)	0.018^***^
Alignment correction (°)	4.2 (±1.7)	5.0 (±2.8)	3.1 (±7.8)	0.342

Varus alignment is displayed as a positive value, valgus alignment is a negative value

*MUKA* medial unicompartmental knee arthroplasty, *TKA* total knee arthroplasty, *ANOVA* one-way analysis of variance, *SD* standard deviation, *BMI* body mass index, *M* male, *F* female, *OA* osteoarthritis, *KL* Kellgren-Lawrence grade, *MC* medial compartment, *LC* lateral compartment, *PFC* patellofemoral compartment

^*^TKA patients included more females when compared to both medial UKA cohorts. No differences were noted between both medial UKA cohorts

^**^TKA patients had more severe OA of the lateral and patellofemoral compartment when compared to medial UKA onlay and inlay patients (all *p* < 0.05). No differences were seen between both medial UKA procedures (*p* > 0.05)

^***^TKA patients had more neutral alignment when compared to medial UKA Inlay patients (*p* < 0.05). No differences were seen between TKA and medial UKA onlay or between medial UKA onlay and medial UKA inlay

One author (A.D.P.) performed all surgeries. Medial UKA surgery was performed using robotic-assistance (MAKO Surgical Corp., Ft. Lauderdale, FL, USA) [43, 62], and patients received a RESTORIS® MCK Medial Inlay or Onlay implant (MAKO Surgical Corp, Ft. Lauderdale, FL, USA). The surgical goal was relative alignment undercorrection in order to prevent OA progression at the contralateral compartment [25, 54, 58, 59]. TKA surgery was performed using computer navigation assistance. Patients received a posterior stabilized Vanguard® Total Knee (Biomet, Warsaw, IN, USA), and the goal of the surgery was postoperative neutral alignment. Cementation was used in all surgeries and the patella was resurfaced in all TKA surgeries (Fig. 1).

WOMAC scores were prospectively collected. WOMAC index is a questionnaire of 24 Likert-scale-based questions and is validated for knee OA [7, 8]. This questionnaire reports overall outcome (all 24 questions) and the three subdomains: pain (five questions), stiffness (two questions), and function (17 questions). The overall score and subdomain scores were indexed with 0 as the worst possible score and 100 as the best possible score. The WOMAC questionnaire was completed by 116 patients at mean 5.1-year follow-up (range 4.0–7.0 years) (no significant difference in follow-up between groups), and 72 of these patients (62%) completed the preoperative questionnaire. Other data collected included age, BMI, gender, OA severity of the medial, lateral, and patellofemoral compartment using the Kellgren-Lawrence score [24], and lower leg alignment using hip-knee-ankle radiographs [39] (Table 1). Institutional Review Board approval was obtained.

Statistical analysis was performed using SPSS Version 21 (SPSS Inc., Armonk, NY, USA). One-way analysis of variance (ANOVA) and Chi-square tests were used to compare baseline characteristics and preoperative WOMAC scores between the three groups with additional post hoc LSD tests. Independent t-tests were used to compare functional outcomes between inlay and onlay medial UKA, between inlay medial UKA and TKA and between onlay medial UKA and TKA. The Chi-square tests were used to compare revision rates between treatments. All tests were two-sided and the difference was considered significant when *p* < 0.05. Sample size calculation showed that 35 patients were needed in every group to show a clinically relevant 10-point difference in WOMAC score with an alpha of 0.05, power of 80%, enrollment ratio of 1:1, and standard deviation of 15.0.

## Results

No differences in patient demographics were found between groups in age and BMI, severity of medial compartment OA, preoperative alignment, or alignment correction. TKA patients were more often females and had more severe OA of the lateral and patellofemoral compartment and neutral postoperative alignment compared to patients undergoing medial UKA while no differences between inlay and onlay medial UKA were detected (Table 1). No significant or clinical relevant preoperative differences in overall outcome or subdomain scores were detected (Table 2).

**Table 2:** Preoperative scores of patients undergoing medial UKA and TKA

	MUKA inlay (*n* = 29)	MUKA onlay (*n* = 16)	TKA (*n* = 27)	ANOVA
	Mean (±SD)	Mean (±SD)	Mean (±SD)	*p* value
WOMAC total	61.8 (±16.1)	54.4 (±14.2)	52.0 (±16.4)	0.065
WOMAC pain	61.2 (±16.3)	55.0 (±16.4)	52.1 (±15.5)	0.103
WOMAC stiffness	49.8 (±18.4)	48.7 (±19.3)	41.8 (±21.0)	0.289
WOMAC function	63.3 (±18.1)	54.7 (±14.1)	53.1 (±18.4)	0.074

*MUKA* medial unicompartmental knee arthroplasty, *TKA* total knee arthroplasty, *ANOVA* one-way analysis of variance, *SD* standard deviation, *WOMAC* Western Ontario and McMaster Universities Arthritis Index, *PCS* Physical Composite Scale score, *MCS* Mental Composite Scale score, *EQ-5D* EurQuol health status questionnaire

**Fig. 2:**
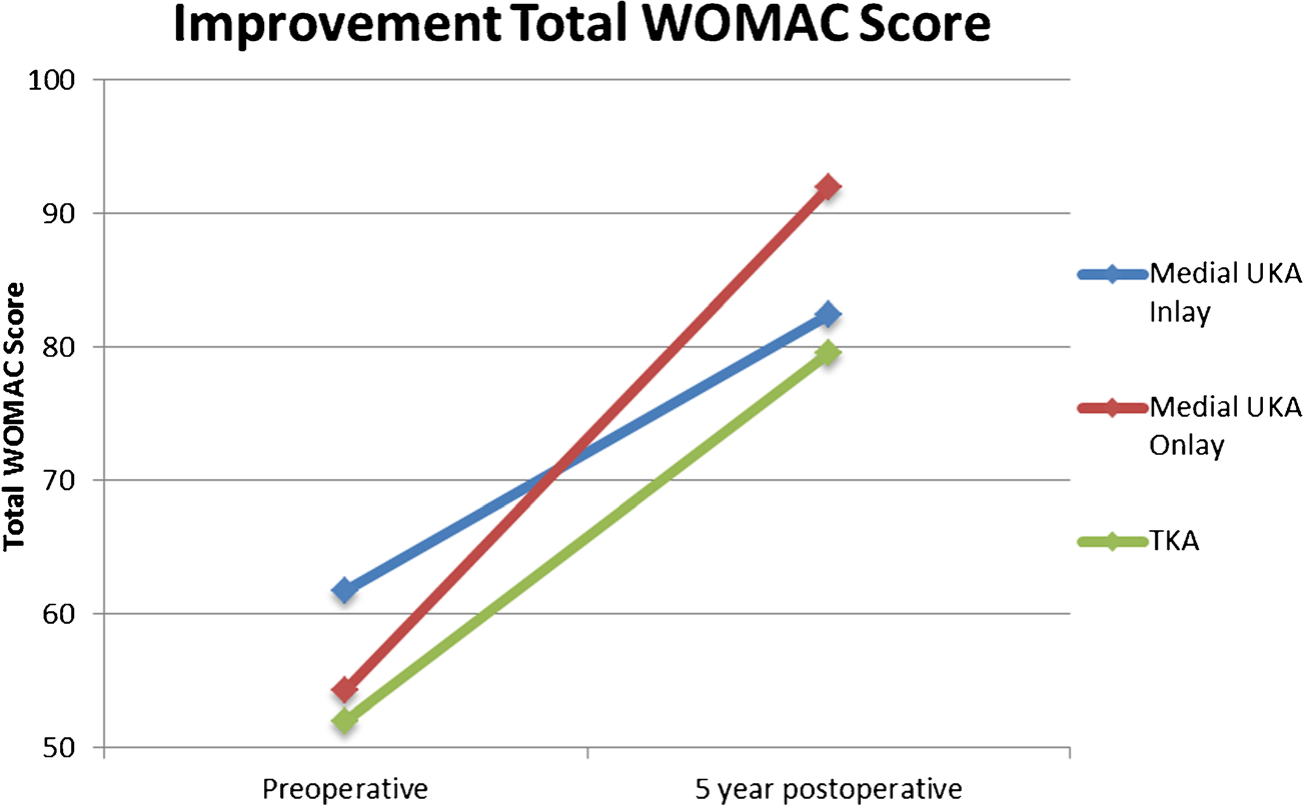
Improvement of the functional outcome scores of the different groups.

**Fig. 3:**
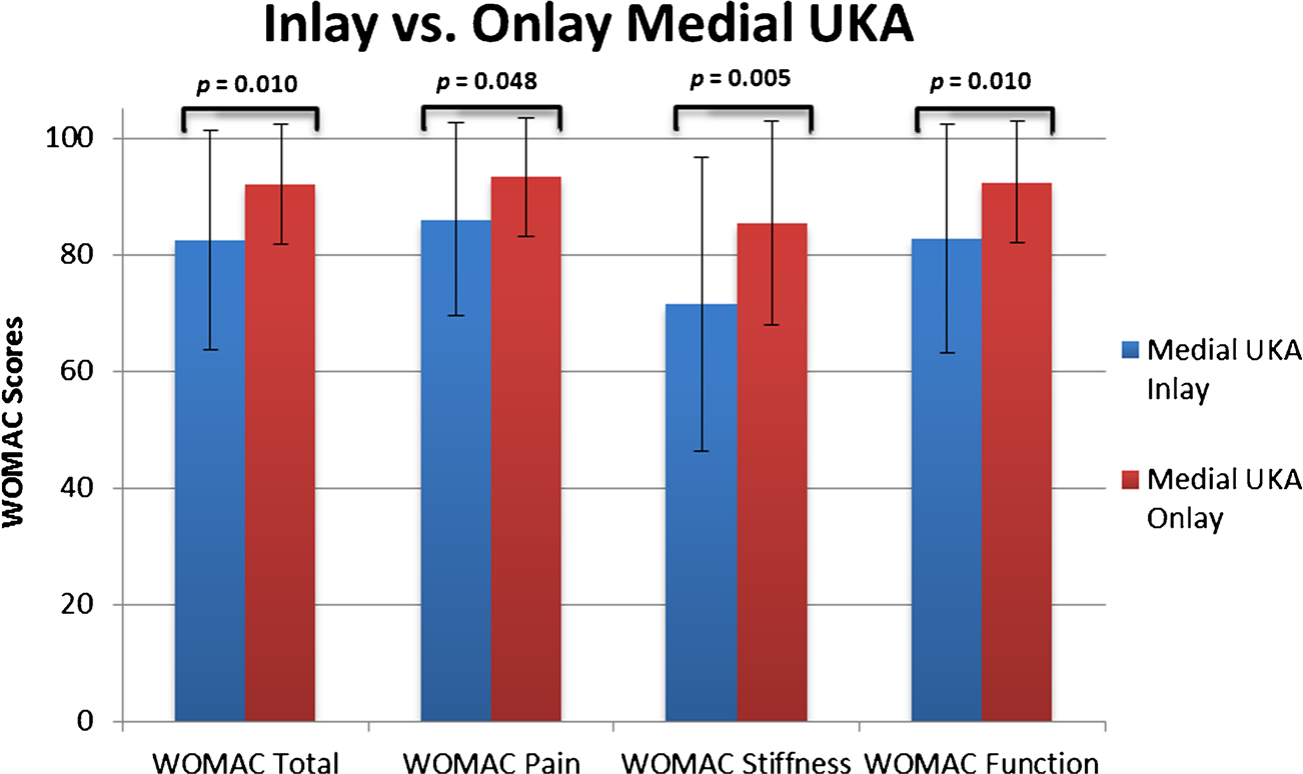
Differences in total WOMAC score and subscores between the medial UKA inlay and onlay.

At mean 5.1-year follow-up, patients undergoing onlay medial UKA reported significant better overall functional outcomes (92.0 ± 10.4 vs. 82.4 ± 18.7, *p* = 0.010) when compared to those of inlay medial UKA. Similarly, patients undergoing onlay medial UKA noted less pain (93.2 ± 10.1 vs. 86.0 ± 16.5, *p* = 0.048), less stiffness (85.6 ± 17.4 vs. 71.6 ± 25.2, *p* = 0.005), and better function (92.4 ± 10.4 vs. 82.6 ± 19.6, *p* = 0.010) (Table 3, Figs. 2 and 3).

**Table 3:** Postoperative scores of patients undergoing medial UKA and TKA

	MUKA inlay (*n* = 36)	MUKA onlay (*n* = 42)	TKA (*n* = 38)	ANOVA
	Mean (±SD)	Mean (±SD)	Mean (±SD)	*p* value
WOMAC total	82.4 (±18.7)	92.0 (±10.4)	79.6 (±18.5)	0.002 ^a,b^
WOMAC pain	86.0 (±16.5)	93.2 (±10.1)	81.3 (±20.2)	0.005 ^a,b^
WOMAC stiffness	71.6 (±25.2)	85.6 (±17.4)	76.8 (±22.1)	0.018 ^a^
WOMAC function	82.6 (±19.6)	92.4 (±10.4)	79.5 (±18.6)	0.002 ^a,b^

*MUKA* medial unicompartmental knee arthroplasty, *TKA* total knee arthroplasty, *ANOVA* one-way analysis of variance, *SD* standard deviation, *WOMAC* Western Ontario and McMaster Universities Arthritis Index

^a^Indicates significant difference (*p* < 0.05) between MUKA inlay and MUKA onlay

^b^Indicates significant difference (*p* < 0.05) between MUKA onlay and TKA

**Fig. 4:**
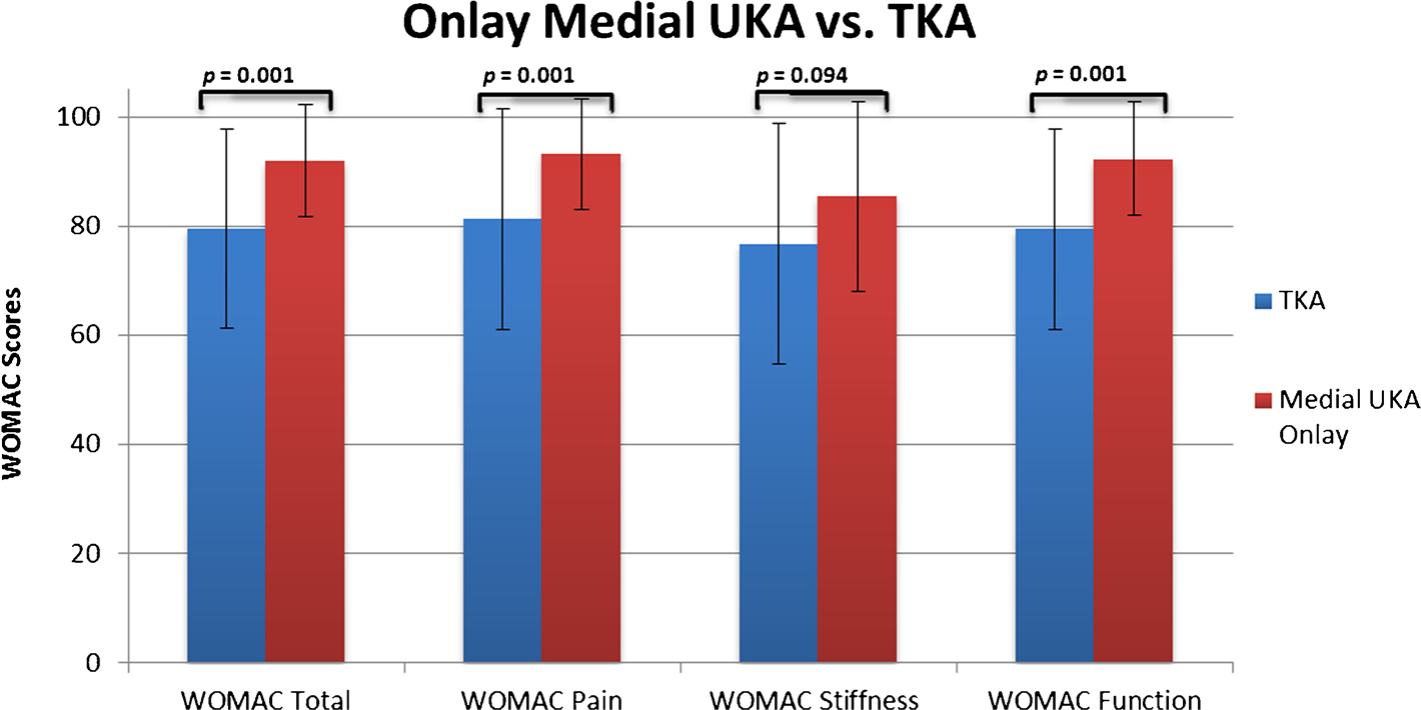
Differences in total WOMAC score and subscores between the medial UKA onlay and TKA.

**Fig. 5:**
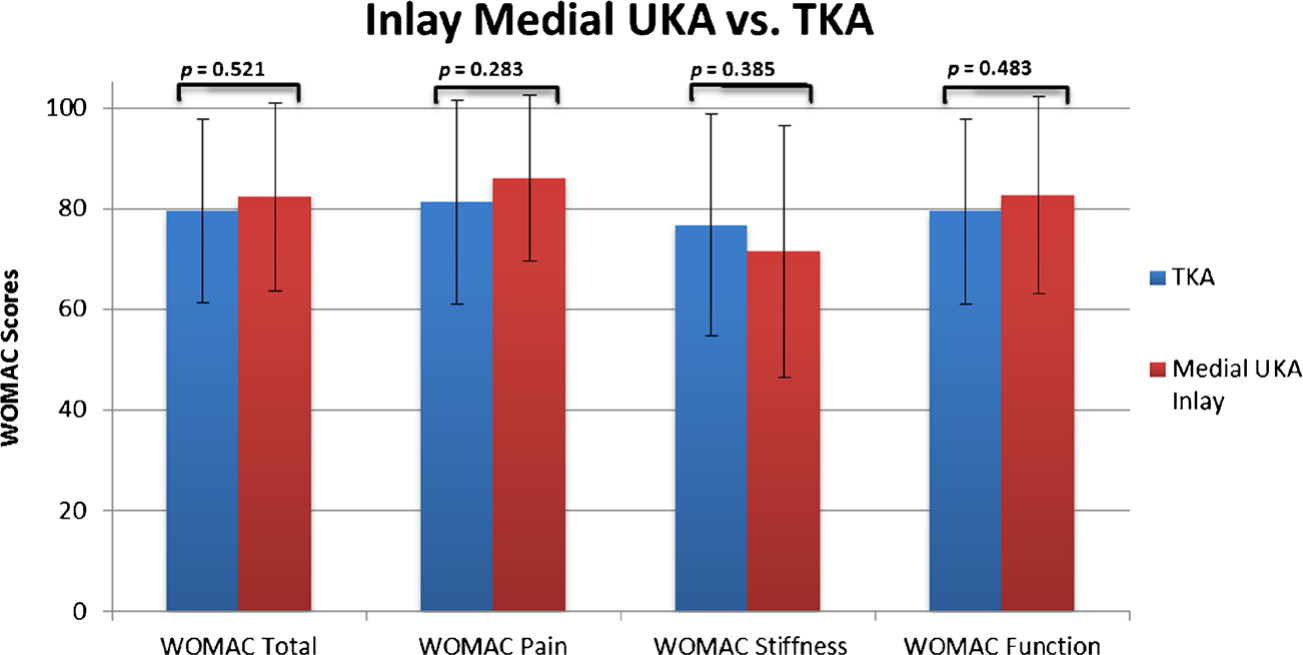
Differences in total WOMAC score and subscores between the medial UKA inlay and TKA.

Significantly better outcomes in onlay medial UKA were noted when compared to those in TKA (92.0 ± 10.4 vs. 79.6 ± 18.5, *p* < 0.001). Patients undergoing onlay medial UKA also reported less pain (93.2 ± 10.1 vs. 81.3 ± 20.2, *p* = 0.001) and better function (92.0 ± 10.4 vs. 79.6 ± 18.5, *p* ≤ 0.001) compared to those of TKA (Table 3, Figs. 2 and 4). Neither significant nor clinically relevant differences could be detected between inlay medial UKA and TKA for overall outcomes or subdomain scores (Table 3, Figs. 2 and 5).

In the inlay medial UKA group, four patients were revised (7.7%), of which three were converted to TKA (two for tibial loosening and one for OA progression) and one was converted to onlay medial UKA due to pain. In the onlay medial UKA group, two patients were converted to TKA (3.4%), both for tibial loosening. Three patients in the TKA group had bearing exchange (5.1%), two for instability and one for an infection. Fewer revisions were noted in the medial UKA onlay group when compared to medial inlay group (*p* = 0.047) but not between other groups (Table 4).

**Table 4:** Revisions and reoperations following medial UKA and TKA procedures

Prosthesis	N	Revisions	Reoperations	All surgeries	Survivorship revisions	Survivorship all surgeries
MUKA inlay	83	4	3	7	95.2%	91.6%
MUKA onlay	194	2	3	5	99.0%	97.4%
TKA	143	3	7	10	97.9%	93.0%
Inlay vs. onlay		0.047	0.280	0.028		
Onlay vs. TKA		0.423	0.073	0.052		
Inlay vs. TKA		0.256	0.652	0.692		

MUKA indicates medial unicompartmental knee arthroplasty; TKA, total knee arthroplasty;

## Discussion

The main finding of this study was that patients undergoing metal-backed onlay medial UKA reported significantly better functional outcomes when compared to patients undergoing all-polyethylene inlay medial UKA at mid-term follow-up. Patients undergoing onlay medial UKA reported better functional outcomes compared with those of TKA while no differences were noted between patients undergoing inlay medial UKA and TKA.

Amalgamating the biomechanical and survivorship data, studies suggest that metal-backed onlay tibial components are superior to all-polyethylene inlay components. This study further demonstrates the superior patient-reported outcomes of metal-backed components over all-polyethylene components. Indeed, to our knowledge, this is the first study demonstrating this finding at mid-term follow-up. Furthermore, this is the first study that compared outcomes of both UKA tibial components with those of TKA. Several limitations are also present in this study. First of all, this is a retrospective study and there was no randomization of the UKA procedures. The senior surgeon switched from the inlay to onlay technique when the onlay prosthesis was clinically released in 2010. Secondly, only 64% of the patients completed the preoperative WOMAC questionnaire and therefore no improvement analysis could be performed. However, preoperatively, no significant or clinical relevant differences were seen between all three groups. Moreover, a trend towards better preoperative outcomes was seen in inlay medial UKA compared to that in onlay medial UKA which would even more dramatically show superiority in functional outcomes of onlay medial UKA (Fig. 2). Finally, robotic-assisted surgery was used for UKA implantation and computer-assisted surgery for TKA implantation and therefore outcomes of this study might not be applicable to manual surgical techniques. However, usage of computer navigation and robot-assistance provided tighter control of other factors that could influence outcomes of knee arthroplasty such as alignment, gap balancing, and component positioning [5, 32, 35, 45, 46, 59] which can highlight the differences in the performance of the implants.

Recently, it has been shown that survivorship of medial UKA at 5-, 10-, and 15-year follow-up is 94, 92, and 89%, respectively [57]. A recent systematic review has shown that aseptic loosening is the most common failure mode in medial UKA [58] while others have shown that this loosening is more common at the tibial side [27]. Therefore, much attention has been paid to tibial designs. Results in the literature regarding onlay or inlay tibial components are mixed as both treatment options have distinct advantages. All-polyethylene inlay components have a thicker polyethylene insert [23], which has the advantage of a decreased risk for revision for polyethylene wear or insert fractures [6]. Metal-backed onlay components require a tibial cut of less depth which has the advantage of relying on the cortical bone while the polyethylene insert can also be replaced in case of polyethylene wear or insert fracture without replacing the tibial or femoral components [33]. Furthermore, biomechanical studies have assessed the stress on the tibial bone with both tibial components designs. Small et al. assessed the maximum shear stress in 12 positions within 3 cm distal to the tibial component and found that the onlay design generates a more favorable strain distribution [49]. Walker et al. also found that inlays generated six times more peak stress than onlay designs, which would increase to 13.5 times when softer bone was present at the tibia [61]. Scott et al. found that inlay implants had a significant increase in damage at the microscopic level compared with onlay implants [47]. It has been suggested that this increased peak stress could result in tibial subsidence or aseptic loosening [3, 66] and pain [48], which could lead to lower survivorship or inferior functional outcomes, respectively. Although several studies have shown that excellent long-term survivorship can be achieved using both onlay [10, 17] and inlay tibial implant designs [34], Zambianchi et al. found an inlay 5-year survivorship of 86% and onlay 5-year survivorship of 100% [66]. Five of these failures were caused by unexplained pain, two by aseptic loosening, two by polyethylene wear, and one for OA progression and one for joint stiffness. Furthermore, Aleto et al. retrospectively reviewed 32 revised UKAs of which 22 were onlay and ten were inlay components [3]. They found that medial tibial subsidence was the failure mode in 87% of failed inlay components while this was only 53% in onlay components. Furthermore, other studies have reported suboptimal results of all-polyethylene tibial designs [9, 12, 22, 37]. These studies may indicate that inlay components have a higher risk of failure due to an increased risk for unexplained pain, tibial subsidence, and aseptic loosening. In this study, a small but signficant difference in revision rate was noted between medial UKA onlay and inlay groups (*p* = 0.047), but studies with larger cohorts are necessary to draw strong conclusions regarding to the revision rates. Because aforementioned studies have also shown differences in revision rates, we expect that more revisions likely occur following a medial UKA inlay procedure in studies with larger cohorts or meta-analysis.

Fewer studies have assessed functional outcomes of inlay and onlay components. Gladnick et al. compared inlay versus onlay components at the 2-year follow-up [19]. Patients in their study underwent, similar to this current study, robotic-assisted UKA surgery, and the authors also reported superior WOMAC scores in patients undergoing metal-backed. Furthermore, they noted a higher revision rate in inlay components compared to that in onlay components although it was non-significant. Reviewing the biomechanical studies and studies reporting survivorship, one might also expect better functional outcomes with metal-backed components at a longer follow-up. In our study, it was indeed noted that metal-backed designs resulted in better outcomes compared to inlay designs at mid-term follow-up. Furthermore, it was noted that patients undergoing onlay medial UKA reported better outcomes, less pain, and better function when compared to those of TKA, while this difference was not seen between inlay medial UKA and TKA. Other studies, however, have failed to show differences at mid-term follow-up. Hutt et al. performed a randomized clinical trial of onlay versus inlay medial UKA implants [22]. The authors reported survivorship at 7-year follow-up of onlay of 94% and inlay of 57%, which was significantly higher. Interestingly, they reported better WOMAC scores in patients undergoing inlay medial UKA at mid-term follow-up, although they did not find any differences in KOOS scores or satisfaction rates. They concluded that reasonable functional results were achieved with both component designs and recommended that inlay medial UKA had unsatisfactory results compared to onlay medial UKA. Heyse et al. performed a subgroup analysis in their mid-term results of fixed-bearing UKA [20]. Interestingly, they found that males (but not females) undergoing inlay medial UKA reported significantly better Knee Society Score (KSS) Function score compared to those of cemented onlay medial UKA. Finally, Hyldahl et al. could not find any short-term differences in Hospital for Special Surgery scores between inlay and onlay designs [23].

Although many studies have assessed functional outcomes following UKA to TKA [2, 4, 11, 15, 16, 21, 29–31, 36, 40, 41, 51, 60, 65, 67], most of these studies are mobile-bearing UKA designs, are mixed onlay and inlay fixed-bearing, or a combination of these. A few studies have compared patient-reported outcomes of fixed-bearing UKA versus TKA [2, 36, 40, 67]. Two studies compared the all-polyethylene St George Sled UKA with TKA [2, 40]. Ackroyd et al. did not find any significant differences in Bristol Knee Scores (BKS) [2], while Newman et al. also could not find any significant difference in BKS between both procedures [40]. Two studies have compared metal-backed onlay UKA with TKA and both found significant better outcomes in UKA patients [36, 67]. Manzotti et al. reported better KSS Function scores in UKA patients [36] while Zuiderbaan et al. found that UKA patients had less joint awareness during activities [67]. These studies suggest that onlay components may have better outcomes than TKA while inlay components are not superior to TKA, similar to what was found in our study.

In conclusion, the results of this study show that superior functional outcomes were reported at mid-term follow-up in patients undergoing metal-backed medial UKA compared to those of all-polyethylene medial UKA. Furthermore, metal-backing medial UKA had superior functional outcomes when compared to TKA while outcomes following all-polyethylene medial UKA were equivalent to TKA. Based on the results of this study and other studies in the literature, we recommended the use of metal-backed onlay tibial components for unicompartmental knee arthroplasty.

## Electronic supplementary material

ESM 1 (PDF 1224 kb)

ESM 2 (PDF 1224 kb)

ESM 3 (PDF 1224 kb)

ESM 4 (PDF 1224 kb)
